# Protective and risk factors of social support for healthcare workers in high-pressure occupational settings

**DOI:** 10.3389/fpsyg.2025.1547777

**Published:** 2025-05-09

**Authors:** Jingtao Gui, Kaiwen Liang, Yahui Yang, Lian Du

**Affiliations:** ^1^Department of Psychiatry, The First Affiliated Hospital of Chongqing Medical University, Chongqing, China; ^2^Gele Mountain Hospital of Chongqing Mental Health Center, Chongqing, China; ^3^Health Management Center, The First Affiliated Hospital of Chongqing Medical University, Chongqing, China; ^4^Key Laboratory of Major Brain Disease and Aging Research (Ministry of Education), Chongqing Medical University, Chongqing, China

**Keywords:** social support, healthcare workers, high-pressure occupational settings, mental health, protective and risk factors

## Abstract

**Background:**

Social support is a critical factor in maintaining the mental health of healthcare workers in high-pressure occupational settings. However, few studies have systematically explored the different types of social support and their influencing factors. This study aims to investigate the current status of social support for healthcare workers under high pressure, along with the related protective and risk factors.

**Methods:**

This cross-sectional study collected data through an online questionnaire involving 625 doctors and nurses from the First Affiliated Hospital of Chongqing Medical University. The Social Support Rating Scale (SSRS) was used to assess levels of social support, including objective support, subjective support, and support utilization. Data analysis was conducted using SPSS 26.0, employing univariate and linear regression analyses to examine gender differences and the impact of various factors on social support.

**Results:**

The study found that the total social support score was increased by factors such as family cohesion, living with others, older age, and professional identity (with doctors achieving higher scores than nurses), while insecure attachment and being an only child reduced the total social support score.

**Conclusion:**

This study reveals the multidimensional impact of various factors on social support for healthcare workers in high-pressure occupational settings. These findings provide a basis for promoting individual mental health and also highlights the need to pay more attention to the mental health of those healthcare workers with poor interpersonal relationships, younger individuals, and nurses in high-pressure environments.

## Introduction

The importance of social support becomes increasingly evident when addressing psychological stress and the unique challenges faced by healthcare workers in high-pressure occupational settings. Social support can be categorized into two primary types: objective support, which refers to tangible or actual assistance, and subjective support, which pertains to emotional or psychological support experienced by individuals. Additionally, the capacity of individuals to utilize social support should also be considered (Xiao, 1999). Social support is widely recognized as a protective factor for mitigating psychological distress and promoting mental well-being.

The buffering model, first proposed by Cobb (1976), explains how social support acts as a barrier to mitigate the stress experienced by individuals. This model suggests that individuals with robust social support networks feel less affected by stress on their health and well-being due to the shielding or “buffering” effects of social support, which includes various forms such as informational support, recognition, emotional support, and practical assistance (Cobb, 1976; Cohen and McKay, 1984). Essentially, social support derived from interpersonal relationships, particularly family relationships, helps alleviate stress levels, which are directly related to an individual’s quality of life and mental health status, such as depression. Ortiz-Calvo et al. (2022) found a negative correlation between resilience, self-perceived social support, and mental health problems after controlling for potential confounding factors. Similarly, Schug et al. (2021) demonstrated that higher levels of social support and an optimistic attitude are associated with lower symptoms of depression and generalized anxiety.

Healthcare workers in high-pressure occupational settings, such as those in China, experience significant psychological stress due to the demanding nature of their roles, which often encompass the simultaneous responsibilities of clinical practice, research, and teaching (Wu et al., 2010; Zhang et al., 2020). In addition to routine pressures, healthcare workers often face crisis events such as public health emergencies (Liu et al., 2020), medical disputes (Sun et al., 2017), and other unforeseen challenges, all of which can significantly impact their mental health and overall well-being (Wu et al., 2010; Zhang et al., 2020). However, it has also been observed that not all healthcare workers develop psychological problems under such circumstances (Alonso et al., 2021; Saragih et al., 2021; Sun et al., 2017; Zhang et al., 2020). The varying outcomes are thought to be possibly related to the significant mediating role of social support (Dong et al., 2022; Hutten et al., 2021).

Despite the recognized importance of social support, previous studies have not systematically investigated the different types of social support, as well as the influencing factors related to various forms of social support (Carter et al., 2023; Khoury et al., 2021). Therefore, this study explored the social support systems of healthcare workers in such environments and examined the protective and risk factors associated with these systems. The findings aim to provide valuable insights for future mental health initiatives designed to support healthcare workers.

## Methods

### Participants and methods

This study utilized a cross-sectional research design to investigate healthcare workers in Chongqing. Data were collected via the “Wenjuanxing” platform^1^ from December 12 to December 30, 2022. This platform enabled us to collect data efficiently from geographically dispersed participants. The initial questionnaire draft was developed based on preliminary studies and expert suggestions from psychiatry professionals. Feedback from healthcare professionals was incorporated to refine the language and content, resulting in the final version.

The survey link and data collection forms were distributed in QR code format across various WeChat workgroups at the First Affiliated Hospital of Chongqing Medical University (CQMU), targeting doctors, nurses, clinical staff, and medical students. However, the study included only data from doctors and nurses, excluding responses from medical students and administrative personnel. Participation was voluntary, with the survey’s first page clearly outlining the study’s background and objectives, emphasizing anonymity and confidentiality. Participants were required to provide informed consent to proceed with the questionnaire, ensuring voluntariness and independence. The survey was programmed to allow only one submission per participant to ensure data accuracy. This study adhered to ethical research guidelines and was approved by the Ethics Committee of the First Affiliated Hospital of Chongqing Medical University (K2023-177).

### Questionnaires

#### Social Support Rating Scale (SSRS)

The Xiao Shuiyuan Social Support Scale (SSRS) was used to assess participants’ social support status. This scale is widely utilized in domestic settings and possesses high reliability and validity. It consists of three dimensions: objective support, subjective support, and support utilization, including 10 items with scores ranging from 0 to 66. Objective support refers to the actual support received in life, subjective support refers to perceived support and satisfaction, and support utilization reflects the individual’s ability to effectively utilize support when needed. Higher scores indicate greater social support.

#### Chinese Family Adaptability and Cohesion Evaluation Scale (FACES II-CV)

The Chinese version of the Family Adaptability and Cohesion Evaluation Scale, Second Edition (FACES II-CV) (Fei, 1991) was utilized to evaluate family functioning. This scale measures family communication, interaction, and emotional connection, as well as the ability to cope with changes through two dimensions: family cohesion and adaptability. Cohesion reflects the emotional connection and support among family members, and adaptability refers to the family’s flexibility and coping abilities in the face of stress and change.

#### Adult Attachment Scale (AAS)

This study utilized the Revised Adult Attachment Scale (RAAS) developed by Collins and Read (1996). Previous research has demonstrated that this scale exhibits good reliability and validity when applied in China (Wu et al., 2004). The scale consists of 18 items rated on a 5-point Likert scale, ranging from 1 (Not at all characteristic of me) to 5 (Extremely characteristic of me), and is divided into three subscales: Anxiety, Comfort with Closeness, and Comfort with Depending on Others. To compare attachment profiles, participants were categorized into their respective attachment styles (secure, preoccupied, dismissing, fearful) based on whether their scores on the dimensions of attachment-related anxiety and avoidance were above or below the scale’s midpoint. This study classified preoccupation, avoidance, and fear patterns as insecure attachment patterns, as each individual’s attachment model was either secure or insecure, and this classification was incorporated into the statistical analysis.

#### Other questionnaires

A series of questionnaires were utilized to collect comprehensive data on participants’ health and well-being, with detailed descriptions provided in our previously published articles (Liang et al., 2024).

##### Basic information and mental health status

A self-designed questionnaire was deployed to collect participants’ demographic information, including age, gender and educational background, and to assess their mental health status.

##### Sleep evaluation

Selected items from the Hamilton Depression Rating Scale (HAMD-24) (Hamilton, 1967) were used to assess participants’ sleep quality.

##### Visual Analogue Scale (VAS)

This tool evaluated participants’ emotional state, physical health, and perceived stress levels on a scale from 0 to 10.

##### Assessment of perceived stress and psychosomatic health

The perceived stress of healthcare workers was assessed using the Visual Analog Scale (VAS). Scores were recorded, and a threshold score of greater than 8 was employed as a criterion to indicate severe stress.

##### Assessment of general psychological health

This evaluation categorized participants’ psychosomatic distress levels based on their reported emotional, somatic, and sleep-related issues.

### Data analysis

Data analysis was conducted by IBM SPSS 26.0 statistical software. Continuous variables were expressed as means ± standard deviations (M ± SD) and compared using two sample *t*-test or one-way analysis of variance. Categorical variables were expressed as frequencies (percentage) [*n* (%)] and compared using non-parametric tests. A *p*-value of less than 0.05 was deemed statistically significant. In this study, linear regression analysis was used to explore the impact of various factors on social support, with a *p*-value of less than 0.05 also considered statistically significant.

## Results

### Gender differences among healthcare workers

In this study, 625 healthcare professionals participated, with three questionnaires excluded due to irregularities in completion, resulting in a valid questionnaire rate of 99.5%. Participants included 378 doctors and 244 nurses, with an average age of 38.97 years (SD 10.03), ranging from 18 to 75 years. In terms of gender distribution, 28% were male (174 individuals) and 72% were female (448 individuals). Most participants held a bachelor’s degree or higher (84.7%) and primarily originated from nuclear family backgrounds (93.2%).

The comparative analysis revealed significant gender-related disparities across several statistical indicators. Profound differences were observed in profession, age, permanent address, and educational background between genders, achieving high statistical significance (*p* < 0.001). Additionally, gender differences in household income levels, perceived stress scores, and emotional state were statistically significant, with *p*-values of 0.006, 0.003, and 0.002, respectively. Males reported higher perceived stress scores and a greater incidence of abnormal emotional state than females. However, gender differences in social support were not statistically significant (Table 1).

**Table 1:** Gender differences in demographic and psychometric characteristics of healthcare workers.

Variables	Total (*n* = 622)	Gender		*t*/ *χ*^2^/*Z*	*p-*value
		Male 174 (28.0%)	Female 448 (72.0%)		
Vocation				133.931	<0.001*
Doctor	378 (60.8%)	169 (44.7%)	209 (55.3%)		
Nurse	244 (39.2%)	5 (2.0%)	239 (98.0%)		
Age	38.97 ± 10.028	41.71 ± 11.030	37.90 ± 9.411	−3.658	<0.001*
Permanent address				16.362	<0.001*
Rural	103 (16.6%)	23 (13.2%)	80 (17.9%)		
County	138 (22.2%)	23 (13.2%)	115 (25.7%)		
Urban	381 (61.3%)	128 (73.6%)	253 (56.5%)		
The only child				2.333	0.127
No	448 (72.0%)	133 (76.4%)	315 (70.3%)		
Yes	174 (28.0%)	41 (23.6%)	133 (29.7%)		
Educational background				−10.756	<0.001*
Else	10 (1.6%)	4 (2.7%)	6 (1.3%)		
Junior college	85 (13.7%)	7 (4.7%)	78 (17.4%)		
Bachelor’s degree	293 (47.1%)	35 (23.6%)	253 (56.5%)		
Master’s degree	103 (16.6%)	25 (16.9%)	68 (15.2%)		
Doctor’s degree	131 (21.1%)	77 (52.0%)	43 (9.6%)		
Primary family				2.453	0.293
Nuclear family	580 (93.2%)	414 (92.4%)	166 (95.4%)		
Blended family	22 (3.5%)	19 (4.2%)	3 (1.7%)		
Single-parent family	20 (3.2%)	15 (3.3%)	5 (2.9%)		
Household income level				−2.723	0.006*
Not good	30 (4.8%)	15 (8.6%)	15 (3.3%)		
Not very good	34 (5.5%)	14 (8.0%)	20 (4.5%)		
Average	462 (74.3%)	123 (70.7%)	339 (75.7%)		
Good	85 (13.7%)	17 (9.8%)	68 (15.2%)		
Very good	11 (1.8%)	5 (2.9%)	6 (1.3%)		
Living with parents during 0–3 years-old				1.619	0.203
No	101 (16.2%)	23 (13.2%)	78 (17.4%)		
Yes	521 (83.8%)	151 (86.8%)	370 (82.6%)		
Living with others				1.041	0.308
No	86 (13.8%)	28 (16.1%)	58 (12.9%)		
Yes	536 (86.2%)	146 (83.9%)	390 (87.1%)		
Adult attachment style				3.049	0.081
Secure	482 (78.4%)	143 (86.2%)	339 (75.7%)		
Insecure	140 (21.6%)	31 (17.8%)	109 (24.3%)		
Social support rate scale					
Objective support	11.25 ± 4.164	11.16 ± 3.600	11.28 ± 4.367	−0.136	0.892
Subjective support	24.00 ± 4.649	24.13 ± 4.443	23.96 ± 4.731	−0.332	0.740
Utilization of support	7.78 ± 1.871	7.61 ± 1.934	7.84 ± 1.845	−1.660	0.097
Family cohesion	71.13 ± 11.164	71.27 ± 9.630	71.08 ± 11.716	−0.322	0.748
Family adaptability	50.95 ± 9.799	51.14 ± 8.528	50.87 ± 10.258	−0.075	0.940
Perceived stress					
Perceived stress scores	5.38 ± 2.533	5.84 ± 2.475	5.21 ± 2.536	−2.976	0.003*
Perceived stress – Threshold 8				1.825	0.177
Normal	487 (78.3%)	130 (74.7%)	357 (79.7%)		
Severe stress	135 (21.7%)	44 (25.3%)	91 (20.3%)		
Sleep state				3.137	0.077
Normal	501 (80.5%)	148 (85.1%)	353 (78.8%)		
Abnormal	121 (19.5%)	26 (14.9%)	95 (21.2%)		
Emotional state				9.777	0.002 *
Normal	413 (66.4%)	99 (56.9%)	314 (70.1%)		
Abnormal	209 (33.6%)	75 (43.1%)	134 (29.9%)		
Physical health state				0.665	0.415
Normal	475 (76.4%)	129 (74.1%)	346 (77.2%)		
Abnormal	147 (23.6%)	45 (25.9%)	102 (22.8%)		
Psychosomatic distress				3.670	0.160
No	336 (54.0%)	85 (48.9%)	251 (56.0%)		
Moderate	238 (38.3%)	77 (44.3%)	161 (35.9%)		
Severe	48 (7.7%)	12 (6.9%)	36 (8.0%)		

*p < 0.05.

### Univariate analysis of social support

A comparative analysis was conducted to assess the impact of various independent variables on social support. Tables 2, 3 indicated significant differences in the total social support score concerning only-child status, primary family, household income level, living with others, attachment style, perceived stress, sleep state, emotional state, psychosomatic distress, age, family cohesion, and family adaptability (*p* < 0.05).

**Table 2:** Univariate analysis of social support in healthcare workers.

Variables	Objective support	*t/F*	*p*	Subjective support	*t/F*	*p*	Utilization of support	*t/F*	*p*	Social support rate scale	*t/F*	*p*
	M ± SD			M ± SD			M ± SD			M ± SD		
Gender		0.323	0.747		−0.412	0.681		1.37	0.171		0.239	0.811
Female	11.28 ± 4.367			23.96 ± 4.731			7.84 ± 1.845			43.08 ± 8.540		
Male	11.16 ± 3.600			24.13 ± 4.443			7.61 ± 1.934			42.90 ± 7.761		
Vocation		−0.642	0.521		−0.228	0.82		−1.791	0.074		−0.854	0.394
Doctor	11.16 ± 3.601			23.97 ± 4.437			7.67 ± 1.871			42.80 ± 7.861		
Nurse	11.38 ± 4.916			24.06 ± 4.969			7.95 ± 1.864			43.39 ± 8.997		
Permanent address		0.317	0.729		3.557	0.029*		1.329	0.265		1.917	0.148
Rural	11.52 ± 3.469			23.74 ± 4.604			7.75 ± 1.713			43.01 ± 8.113		
County	11.10 ± 6.122			23.18 ± 5.271			7.57 ± 1.922			41.85 ± 10.420		
Urban	11.23 ± 3.401			24.37 ± 4.384			7.87 ± 1.892			43.46 ± 7.463		
The only child		2.681	0.008*		3.579	0.000*		0.318	0.75		3.226	0.001*
No	11.49 ± 4.479			24.42 ± 4.505			7.79 ± 1.877			43.70 ± 8.407		
Yes	10.63 ± 3.144			22.94 ± 4.857			7.74 ± 1.861			41.32 ± 7.782		
Educational background		1.123	0.344		1.64	0.163		1.63	0.165		1.945	0.101
Else	10.30 ± 3.713			24.80 ± 4.264			6.90 ± 1.449			42.00 ± 7.165		
Junior college	10.59 ± 3.160			23.40 ± 5.164			7.46 ± 1.790			41.45 ± 7.738		
Bachelor’s degree	11.38 ± 4.822			24.00 ± 4.957			7.88 ± 1.878			43.26 ± 9.248		
Master’s degree	11.00 ± 3.506			23.47 ± 4.043			7.68 ± 1.854			42.15 ± 7.500		
Doctor’s degree	11.64 ± 3.599			24.76 ± 3.958			7.92 ± 1.930			44.31 ± 6.954		
Primary family		3.18	0.042		4.033	0.018*		2.45	0.087		5.407	0.005*
Nuclear family	11.36 ± 4.184			24.13 ± 4.590			7.82 ± 1.874			43.31 ± 8.264		
Blended family	9.77 ± 3.854			23.00 ± 5.318			7.59 ± 1.681			40.36 ± 8.572		
Single-parent family	9.60 ± 3.347			21.35 ± 4.902			6.90 ± 1.861			37.85 ± 7.936		
Household income level		1.158	0.328		4.808	0.001*		1.654	0.159		3.53	0.007*
Not good	10.03 ± 3.596			22.50 ± 4.273			7.03 ± 1.564			39.57 ± 6.500		
Not very good	11.47 ± 3.017			23.50 ± 3.527			7.68 ± 1.628			42.65 ± 6.035		
Average	11.20 ± 4.320			23.81 ± 4.663			7.79 ± 1.826			42.80 ± 8.457		
Good	11.87 ± 3.835			25.24 ± 4.888			7.98 ± 2.166			45.08 ± 8.548		
Very good	11.09 ± 4.182			28.09 ± 2.166			8.27 ± 2.494			47.45 ± 7.202		
Living with parents during 0–3 years-old		−0.52	0.958		−2.497	0.013*		0.305	0.761		−1.347	0.178
No	11.23 ± 6.525			22.95 ± 4.683			7.83 ± 2.069			42.01 ± 9.880		
Yes	11.25 ± 3.538			24.21 ± 4.620			7.77 ± 1.833			43.23 ± 7.982		
Living with others		−3.585	0.000*		−2.966	0.004*		−0.56	0.576		−3.419	0.001*
No	9.77 ± 3.593			22.43 ± 5.420			7.66 ± 2.123			39.86 ± 9.447		
Yes	11.49 ± 4.203			24.26 ± 4.468			7.80 ± 1.829			43.54 ± 8.022		
Adult attachment style		6.849	0.000*		9.305	0.000*		7.145	0.000*		10.439	0.000*
Secure	11.84 ± 4.217			24.88 ± 4.343			8.04 ± 1.853			44.76 ± 7.789		
Insecure	9.20 ± 3.237			20.99 ± 4.412			6.88 ± 1.647			37.06 ± 7.302		
Perceived stress – Threshold 8		1.225	0.221		2.871	0.004*		1.679	0.094		2.596	0.010*
Normal	11.36 ± 4.371			24.28 ± 4.600			7.85 ± 1.828			43.48 ± 8.374		
Severe stress	10.86 ± 3.299			22.99 ± 4.704			7.54 ± 2.020			41.39 ± 7.952		
Sleep state		2.121	0.000*		3.684	0.000*		2.85	0.005*		3.772	0.000*
Normal	11.42 ± 4.249			24.34 ± 4.561			7.88 ± 1.855			43.64 ± 8.308		
Abnormal	10.53 ± 3.724			22.62 ± 4.775			7.35 ± 1.883			40.50 ± 7.929		
Emotional state		−0.66	0.947		4.346	0.000*		3.71	0.000*		3.21	0.001*
Normal	11.24 ± 3.506			24.57 ± 4.662			7.98 ± 1.871			43.79 ± 8.043		
Abnormal	11.26 ± 5.237			22.88 ± 4.425			7.39 ± 1.816			41.54 ± 8.679		
Physical health state		−0.625	0.106		2.744	0.006*		1.8	0.072		1.618	0.106
Normal	11.19 ± 3.537			24.29 ± 4.737			7.85 ± 1.884			43.33 ± 8.124		
Abnormal	11.44 ± 5.752			23.09 ± 4.243			7.54 ± 1.814			42.06 ± 8.895		
Psychosomatic distress		2.03	0.132		13.361	0.000*		7.982	0.000*		9.927	0.000*
No	11.29 ± 3.521			24.77 ± 4.621			8.02 ± 1.900			44.08 ± 8.077		
Moderate	11.42 ± 4.929			23.39 ± 4.555			7.60 ± 1.783			42.40 ± 8.446		
Severe	10.10 ± 4.091			21.65 ± 4.123			7.02 ± 1.828			38.77 ± 7.883		

*p < 0.05.

**Table 3:** Univariate analysis of social support in healthcare workers (continuous variables).

Variables	Objective support		Subjective support		Utilization of support		Social support rate scale	
	*r*	*p*	*r*	*p*	*r*	*p*	*r*	*p*
Age	0.078	0.051	0.259^**^	0.000	0.034	0.394	0.191^**^	0.000
Family cohesion	0.331^**^	0.000	0.476^**^	0.000	0.295^**^	0.000	0.498^**^	0.000
Family adaptability	0.281^**^	0.000	0.483^**^	0.000	0.284^**^	0.000	0.474^**^	0.000
Perceived stress scores	−0.008	0.846	−0.178^**^	0.000	−0.127^**^	0.000	−0.132^**^	0.001

**p < 0.05.

For objective support, significant differences were observed in variables such as only-child status, primary family, living with others, attachment style, sleep state, family cohesion, and family adaptability (*p* < 0.05).

Regarding subjective support, factors such as permanent address, only-child status, primary family, household income level, living with parents between ages 0–3, living with others, attachment style, perceived stress, sleep state, emotional state, physical health state, psychosomatic distress, age, family cohesion, family adaptability, and perceived stress exhibited statistical differences (*p* < 0.05).

In terms of support utilization, significant differences were identified in variables such as attachment style, sleep state, emotional state, psychosomatic distress, family cohesion, family adaptability, and perceived stress (*p* < 0.05) (Tables 2, 3).

### Regression analysis of social support

#### Total social support score

A linear regression analysis was performed to evaluate comprehensively the variables associated with social support, constructing a regression model. The *R*-squared value was 0.358, indicating that the model accounted for 35.8% of the variance in the data. In this model, higher family cohesion, living with others, older age, and doctor status (as opposed to a nurse) positively increased the level of social support. Conversely, insecure attachment styles and only-child status decreased social support levels (Tables 4, 5, Model 1).

**Table 4:** Regression model summary.

Model	*R*	*R* ^2^	Adjusted *R*^2^	Standard error of the estimate	*F*	*p*
Model 1	0.599	0.358	0.351	6.704	49.017	0.000
Model 2	0.412	0.170	0.163	3.810	25.164	0.000
Model 3	0.597	0.356	0.347	3.758	37.591	0.000
Model 4	0.375	0.141	0.134	1.742	20.205	0.000

Model 1 Predictor variable: (constant), family cohesion, adult attachment style, the only child, living with others, educational background = Junior college, age, vocation; Dependent variable: total social support score.

Model 2 Predictor variable: (constant), family cohesion, adult attachment style, living with others, the only child, emotional state; Dependent variable: objective support.

Model 3 Predictor variable: (constant), family adaptability, adult attachment style, age, psychosomatic distress = severe, the only child, family cohesion, living with others, permanent address = county, psychosomatic distress = moderate; Dependent variable: subjective support.

Model 4 Predictor variable: (constant), family cohesion, adult attachment style, emotional state, educational background = junior college, vocation; Dependent variable: support utilization.

**Table 5:** Linear regression analysis of factors influencing healthcare workers’ social support.

Predictor variable	Unstandardized coefficient		Standardized coefficient	*t*	*p*	Collinearity statistics	
	*B*	Standard error	Beta			Tolerance	VIF
Model 1							
(Constant)	16.996	2.304		7.377	0.000		
Family cohesion	0.299	0.026	0.401	11.693	0.000	0.888	1.127
Adult attachment style	−5.226	0.673	−0.262	−7.769	0.000	0.916	1.092
The only child	−2.052	0.623	−0.111	−3.293	0.001	0.924	1.082
Living with others	2.580	0.785	0.107	3.288	0.001	0.985	1.015
Educational background = Junior college	−2.367	0.813	−0.098	−2.911	0.004	0.927	1.079
Age	0.073	0.029	0.087	2.494	0.013	0.849	1.177
Vocation	1.279	0.586	0.075	2.181	0.030	0.882	1.134
Model 2							
(Constant)	2.774	1.159		2.394	0.017		
Family cohesion	0.107	0.014	0.286	7.391	0.000	0.900	1.111
Adult attachment style	−1.840	0.382	−0.185	−4.813	0.000	0.915	1.092
Living with others	1.499	0.445	0.124	3.368	0.001	0.989	1.011
The only child	−0.813	0.340	−0.088	−2.389	0.017	0.999	1.001
Emotional state	0.702	0.330	0.080	2.132	0.033	0.963	1.038
Model 3							
(Constant)	11.371	1.243		9.145	0.000		
Family adaptability	0.112	0.030	0.236	3.766	0.000	0.268	3.729
Adult attachment style	−2.280	0.382	−0.205	−5.962	0.000	0.89	1.124
Age	0.066	0.016	0.143	4.163	0.000	0.888	1.127
Psychosomatic distress = Severe	−1.664	0.596	−0.096	−2.791	0.005	0.898	1.114
The only child	−1.072	0.353	−0.104	−3.035	0.003	0.904	1.106
Family Cohesion	0.069	0.026	0.166	2.624	0.009	0.264	3.788
Living with others	0.991	0.444	0.074	2.234	0.026	0.969	1.032
Permanent address = County	−0.915	0.374	−0.082	−2.445	0.015	0.94	1.064
Psychosomatic distress = Moderate	−0.722	0.324	−0.076	−2.230	0.026	0.916	1.091
Model 4							
(Constant)	5.003	0.537		9.313	0.000		
Family cohesion	0.038	0.007	0.227	5.780	0.000	0.901	1.110
Adult attachment style	−0.835	0.175	−0.187	−4.783	0.000	0.917	1.091
Emotional state	−0.349	0.152	−0.088	−2.291	0.022	0.942	1.062
Educational background = Junior college	−0.511	0.210	−0.094	−2.427	0.016	0.934	1.071
Vocation	0.316	0.149	0.083	2.125	0.034	0.924	1.082

#### Objective support

For the regression analysis of objective support, the *R*-squared value was 0.170, indicating that the model explained 17% of the variance in objective support scores. In this model, higher family cohesion, living with others, and a normal emotional state (as opposed to abnormal emotional state) increased objective support scores. In contrast, insecure attachment styles and only-child status decreased objective support scores (Tables 4, 5, Model 2).

#### Subjective support

In the analysis of subjective support, the R-squared value was 0.356, indicating that the model explained 35.6% of the variance in subjective support. Here, higher family adaptability and cohesion, older age, and living with others increased subjective support scores. Conversely, insecure attachment styles and only-child status decreased subjective support scores (Tables 4, 5, Model 3).

#### Support utilization

Finally, the *R*-squared value for the regression analysis of support utilization was 0.141, indicating that the model explained 14.1% of the variance in support utilization. In this model, higher family cohesion and doctor status (as opposed to a nurse) increased support utilization. Insecure attachment styles and abnormal emotional state decreased support utilization (Tables 4, 5, Model 4).

## Discussion

Previous studies on healthcare workers’ mental health in challenging work contexts have mainly focused on anxiety, depression, and sleep quality, with relatively little exploration of social support. Social support, however, is a multidimensional construct that encompasses various forms, including subjective support, objective support, and support utilization. Despite its recognized importance, these specific dimensions of social support have been insufficiently studied, which somewhat limits our comprehensive understanding of healthcare workers’ mental health.

This study compared gender differences among healthcare workers. The results revealed significant gender differences in profession, age, permanent address, educational background, household income level, perceived stress scores, and abnormal emotional state. In this study’s sample, the nurse group was predominantly female, and the average age of females was lower than that of males, which could contribute to occupational and age differences. Differences in educational background may arise from varying tendencies between males and females in accessing higher education opportunities and professional choices (White et al., 2012). Generally, males are more inclined to choose technical medical specialties, while females tend to choose nursing and similar professions. Female healthcare workers may bear greater economic pressure (Jolly et al., 2014), particularly when shouldering family responsibilities, such as caring for children or the elderly. This may restrict their career choices and development opportunities, contributing to gender differences in family economic status. Additionally, gender differences in perceived stress and abnormal emotional states differ from previous studies (Pappa et al., 2020), potentially because male healthcare workers might have taken on more high-risk tasks or leadership roles in these challenging work contexts, roles typically accompanied by higher stress and a sense of responsibility (Morgan et al., 2022). In many cultures, men are expected to appear strong and refrain from expressing emotions. This social expectation may lead males to internalize emotions when facing pressure rather than seeking support or expressing feelings. In the linear regression analysis conducted in this study, several factors were identified as influencing levels of social support. Specifically, higher family cohesion, living with others, older age, and doctor status (as opposed to a nurse) positively increased the level of social support. Conversely, insecure attachment styles and only-child status decreased social support levels. These findings highlight the significance of social support and family intimacy as protective factors for mental health (Wright and Davidson Mhonde, 2022). Furthermore, existing literature corroborates the notion that cohesive families can provide enhanced social support and encouragement (Farrell and Barnes, 1993; Souri and Ashoori, 2015; Wright and Davidson Mhonde, 2022). The increased social support reported by individuals living with others can be explained by social interaction theory in high-pressure occupational settings, living with family members or roommates provides opportunities for emotional exchange, practical assistance, and shared coping strategies, which collectively enhance the sense of social support and resilience (Cohen and McKay, 1984; Halbesleben, 2006; Holt-Lunstad et al., 2010). Additionally, age significantly influences social support levels. Previous literature suggests that as individuals grow older, they often accumulate more social resources and support networks, allowing them to enjoy higher social support (Kröner and Müller, 2022). The phenomenon of doctors having higher social support than nurses possibly relate to professional characteristics and work environments. Nurses interact more with patients in their work, and irregular working hours in demanding work contexts may lead to less perceived support (Pappa et al., 2020). In contrast, doctors often have more extensive professional networks established through their education and professional experiences, which can provide strong support when needed. Compared to doctors, nurses may face limitations in social status and resources, resulting in lower reported levels of social support. The link between insecure attachment styles and lower social support levels is consistent with previous research (Adar et al., 2022; Florian et al., 1995). Individuals with insecure attachment styles may encounter difficulties in forming and maintaining intimate relationships, thus limiting their sources of social support. They may be more prone to handling social relationships with avoidance or anxiety, leading to lower social support levels. Finally, the lower social support among only children may be partly due to the absence of sibling interactions, which help develop skills for forming external social relationships. Individuals who grow up without sibling interactions may lack certain skills for establishing and maintaining external social relationships.

This study illustrates that social support is significantly affected by a variety of factors. The primary determinants influencing social support and its different dimensions include family relationships, living environment, professional roles, age, and emotional state. Traditional research on social support often focuses solely on overall levels, overlooking its complexity. By separately analyzing objective support, subjective support, and support utilization, this study reveals the roles and influencing factors of different dimensions of social support in challenging work contexts. Decomposing social support into different dimensions allows for a more detailed identification of which factors significantly influence specific types of support. Specifically, family cohesion has a significant positive impact on subjective support, objective support, and support utilization, indicating that close family relationships play a central role in the perception, acquisition, and utilization of social support. Living with others has a significant positive effect on both subjective support and objective support, suggesting that a shared living environment can enhance the perception of emotional support and provide tangible resources. The impact of emotional state varies across dimensions: normal emotional state significantly improves objective support, while abnormal emotional state may hinder support utilization. Professional identity has a significant effect on support utilization, indicating that occupational resources and social status play an important role in the effective use of support resources. Only-child status has a significant negative impact on both subjective support and objective support, highlighting the importance of family structure in shaping the perception and acquisition of social support. However, the effect of only-child status on support utilization is not significant, which may reflect that support utilization is more influenced by other factors, such as professional identity and emotional state, rather than family structure. Although objective support is visible and tangible, subjective support—perceived support—often has a more direct impact on mental health and behavior. Support utilization, a relatively understudied dimension, considers how individuals effectively use resources once obtained, which is crucial for understanding the practical utility of social support.

Despite its findings, this study has several limitations. Firstly, the study sample was limited to healthcare workers from a single hospital, potentially introducing regional bias that may not reflect broader circumstances. To enhance representativeness and generalizability, future studies should expand the sample size and include more diverse populations. Secondly, the cross-sectional design limits exploration of causal relationships between variables. To better explore the interactions between variables, future research should consider employing longitudinal designs to track variable changes over time and reveal causal relationships. Furthermore, reliance on self-reports may introduce recall and social desirability biases, reducing result accuracy. To address this limitation, future research could consider incorporating multiple data collection methods, such as observations, interviews, and physiological measurements, to cross-verify the authenticity and reliability of self-reported data. Finally, despite controlling for several confounding variables, unmeasured factors may still influence results. For example, differences in work nature among departments and recent significant family events might have potential impacts on study results. Therefore, future data collection processes should enhance the gathering and analysis of participants’ background information to adequately consider and control these potential variables during analysis.

## Conclusion

This study found that family cohesion, living with others, and older age increased social support levels, whereas insecure attachment and only-child status decreased social support. Future mental health support for healthcare workers should take into account gender, age, family background, and occupational characteristics to design personalized psychological support plans. In particular, professional psychological counseling and support services are recommended for managing perceived stress and negative emotions. At both hospital and community levels, support groups and resource networks should be established, especially providing more professional support and development opportunities for nurses to enhance their social support levels. Additionally, mental health education and relationship management training should be implemented to help healthcare workers develop more secure attachment styles, thereby improving their psychological resilience and sense of social support in high-pressure occupational settings.

## Data Availability

The datasets used and/or analyzed during the current study available from the corresponding author on reasonable request.

## Footnotes

^1^ https://www.wjx.cn
